# Severe early childhood caries and social determinants in three-year-old children from Northern Thailand: a birth cohort study

**DOI:** 10.1186/s12903-015-0093-8

**Published:** 2015-09-14

**Authors:** Karl Peltzer, Aroonsri Mongkolchati

**Affiliations:** ASEAN Institute for Health Development, Madidol University, Salaya, Phutthamonthon, 73170 Nakhonpathom Thailand; Department of Psychology, University of the Free State, Bloemfontein, 9300 South Africa; HIV/AIDS/STI/and TB (HAST), Human Sciences Research Council, Private Bag X41, Pretoria, 0001 South Africa

**Keywords:** Severe early childhood caries, Preschool children, Social determinants, Thailand

## Abstract

**Background:**

The purpose of this study was to investigate the prevalence and social risk factors of severe early childhood caries in three-year-old children in Northern Thailand, using a birth-cohort study

**Methods:**

The data utilized in this study were from the prospective cohort study of Thai children (PCTC) from the 28 to 38 weeks gestational age until the children reached the age of 36 months (*N* = 597) in Mueang Nan district, Northern Thailand. Questionnaires were administered at different time points and dental examination was conducted at the age of 3 years of the child.

**Results:**

44.1 % of the 3 year old children had S-ECC. In multivariate logistic regression analysis, environmental factors (the use of rain or well water as drinking water, no schooling of mother of child, being male), and risk behaviour (sleeping with a bottle at 30 months) were associated with S-ECC. Further, in bivariate analysis, psychological distress in the mother, lack of spousal relationship support, suckle to sleep when going to bed, introduction of soft drinks at 12 months, having had more frequently sweet food, and less than daily tooth brushing before 30 months were associated with S-ECC.

**Conclusions:**

A very high rate of S-ECC was observed, and oral health may be influenced by social factors.

## Background

“Severe early childhood caries (S-ECC) affects infants and children. It is infectious, has a multifactor etiology and fast development, starting soon after dental eruption. Due to the presence of local sociocultural risk factors, it must be regarded as a symptom of alteration in the child’s health and lack of adequate care.” ([1], p.295) Globally, there is a lack of studies investigating the prevalence and socio-environmental and behavioural risk factors of S-ECC in pre-school aged children [2], and there is little information available in Thailand about the prevalence and socio-environmental and behavioural risk factors of S-ECC in pre-school aged children. Identification of characteristics associated with children’s risk of developing S-ECC may improve preventive strategies.

The prevalence of S-ECC in pre-school age children ranged from 0.8 % (6–71 months) in Nigeria [3], 2.7 % (36–71 months) in Italy [4], 6.5 % (3 year-olds) in Lithuania [5], 9.5 % (3–5 years) in Germany [2], 17.5 % (3–5 years old) in Trinidad [6], 27 % (2 year-olds) in a sample of African Americans [7], 31.1 % (5–6 years) in Ajman, United Arab Emirates [8], 36 % (35–71 months old) in a health facility in Brazil [9], 46 % (2–6 years) in the Inuvik region, Northwest Territories, Canada [10], and 56 % (mean age 42 months) in Cambodia [11].

Factors associated with S-ECC have been identified in terms of 1) *environmental risk factors*, including lack of community water fluoridation [10], Body Mass Index (BMI) overweight [12], and maternal caries experience [11]; 2) *sociocultural risk factors*, including low socioeconomic status [8, 10, 13, 14], and being a single working mother [15]; 3) *risk behaviours*, including feeding and eating patterns such as excessive sugar intake [13, 14, 16, 17]; high snack consumption level [8, 13], breastfeeding ≥7 times daily [13], night-time breast-feeding [9], breastfeeding for more than 12 months [2, 4]; improper infant bottle-feeding habits (use of the nursing bottle in bed [2]; bottle use for liquids other than milk [13]; sleeping with a bottle containing carbohydrates during the night [4]; use of a bottle at night as a substitute for the pacifier and its use on demand during the day [9, 18]; Low-frequency toothbrushing and improper toothbrushing methods [4, 19], start of tooth brushing after the first anniversary [2] and 4) *use of dental services* [2] and those who utilised dental services only when they had a problem [8]. In this study, we explore associations between environmental factors, sociocultural factors, risk behaviours during pregnancy and the child’s early life, and the S-CCC of 3-year-old children in Northern Thailand using longitudinal data. Based on previous studies, it was hypothesized that socio-environmental factors and risk behaviours during pregnancy and the child’s early life were associated with S-ECC.

## Method

### Sample and procedure

The data utilized in this study were from the prospective cohort study of Thai children (PCTC) (Data are available from http://pctc.damus.in.th/damus/), which is an observational, community-based study designed to follow all foetuses from the 28 to 38 weeks gestational age until the children reached the age of 36 months [20]. A sample of the Thai population was achieved in four cohort from a selected district in each of the four regions in Thailand [20]. All pregnant women who resided or planned to bring up their children in the four selected districts and who expected to give birth during the defined period of 1 year were registered [20]. The women were identified in the beginning of the third trimester by a community survey or by retrieval from registers of antenatal care clinics [20]. The children who were eligible for the PCTC project with parental consent during pregnancy were sampled for inclusion in the present study and the time of measurement was every 6 months. Questionnaires were interviewed-administered at participants’ homes, or at health facilities where parents took their 6 month old infants for immunizations [20, 21]. Dental exam data was collected in three sites, and from one site in Northern Thailand data were available for this study. Therefore, the present study included a sub-sample of children from Mueang Nan district in Nan province, Northern Thailand. In the study area 13 % of the population are mountain-dwelling tribal people [20]. Buddhism is the predominant faith (84 %) and most people work in businesses and small farms, sales, services or labour [20]. There is a lower-than-standard level of fluoride in the community drinking water [21]. Dental care is only available at the district hospital. In all, 783 infants were recruited, which was a response rate of 99 % from all contacted families and for 597 children dental caries data were available at the age of 36 months, which is a response rate of 76.2 %. This study was approved by the National Ethics Committee of the Ministry of Public Health of Thailand. All families were clearly informed of all the study procedures and possible risk before signing the consent form [22].

### Measures

#### Dental caries

The outcome variables were the number of decayed, filled and missing teeth (dmft) and surfaces (dmfs) assessed when the children were 3 years of age. Three trained and calibrated examiners in a field setting examined dental caries. The examiners evaluated dental caries by using decayed, filled and missing primary teeth (DMFT) index and surfaces (DMFS) indices following the World Health Organization [23] criteria for dental caries diagnosis. The dental status of the examined surfaces were classified as follows: U = Unerupted surface, no part of the surface emerges to the oral cavity; S = Normal enamel surface/texture and no restoration; D1 = Initial caries/acries limited in enamel, the lesion demonstrates whitish/yellowish opaque with/without mico-cavity but no softened floor/wall; D2 = Caries to dentine, cavitated lesion is seen to extend beyond enamel that certainly catches the probe with softened floor or wall of undermined enamel; D3 = Caries involving pulp; a deep lesion with probable pulpal involvement or deep lesion with present/history of spontaneous pain/swelling/fistula opening [23]. The outcome of this study was S-ECC, defined as ≥1 cavitated, missing or filled smooth surfaces in primary maxillary anterior teeth, or decayed, missing or filled surface (dmfs) values ≥4 [24].

#### Environmental risk factors

*Anthropometric measurements* were assessed according to recumbent length and were measured in all children at 6 and 36 months of age using a graduate board with a fixed headboard and movable footboard (1 m/0.1 cm) [22].

*Infant’s birth weight* was assessed after birth.

*Tobacco use of the mother* and *history of dental cavitation(s) of the mother* were assessed at 28^th^ to 38^th^ week of pregnancy by self-report; and *environmental tobacco use* and the *source of the type of household drinking water* were assessed at 6 months of the study infant [22].

### Sociocultural risk factors

*Sociodemographic information* obtained at 28^th^ to 38^th^ week of pregnancy included parity, parental age, religion, education, household income, family size, birth order, and marital status. The study infant’s sex was recorded after birth.

#### Risk behaviours

Information concerning *infant feeding practice*s such as breast, formula, and complementary feeding was assessed in the diary developmental record from birth to 12 months and 30 months of age [22]. One sweet food consumption index was created with five items, sweet jelly, sweet drink, sweet candy, sweet Thai dessert and snack consumption at 30 months. Cronbach alpha was 0.68 for this sweet food consumption index of this sample.

*Tooth brushing behaviou*r was assessed at 12 and 26 months of the study child.

*Use of oral services* was assessed at 30 months of the study child.

### Data analysis

Data were analysed using the SPSS software package (PASW Statistics 21, IBM Company, 2013). Descriptive statistics were used to describe the sample. We used univariate logistic regression, followed by multivariate backward conditional logistic regression to obtain adjusted odds ratios (AOR) and associated 95 % confidence intervals. All variables with a univariate test *P* value ≤ 0.25 were considered for inclusion in the multiple logistic regression models. The level of statistical significance was a two-sided *p* value < 0.05. Variance inflation factor (VIF) and tolerance values for each model indicate multicollinearity was not a concern in any of the multivariate analyses.

## Results

### Sample characteristics

The total sample size of children recruited from Mueang Nan district with dental caries data was 597. The prevalence of children with severe early childhood dental caries (S-ECC) was 263 (44.1 %) at 36 months. The prevalence of ECC was 68.5 %. Further sample characteristics are described in Tables 1 and 2.

**Table 1:** Sample characteristics of the overall sample (*n* = 597) and S-ECC sub-sample (*n* = 263)

Variables	All	S-ECC
	*N* = 597 (%) or M (SD)	*N* (%) or M (SD)
Environmental risk factors	*N* (%)	*N* (%)
Drinking water in household		
Bottled	211 (35.4)	88 (41.7)
Pipe	138 (23.2)	56 (40.6)
Rain	41 (6.9)	22 (53.7)
Well or other	206 (34.6)	96 (46.6)
Weight at birth		
≥ 2500 g	529 (92.0)	234 (44.2)
< 2500 g	46 (8.0)	20 (43.5)
Smoking during pregnancy	2 (0.4)	0
Secondary smoke (at 1 year)	158 (26.5)	76 (48.1)
Mother with dental cavitation(s) at baseline	332 (66.5)	151 (45.5)
	M (SD)	M (SD)
Height in cms at 6 months [M, SD]	64.8 (2.6)	64.9 (2.4)
Body Mass Index (BMI) score at 3 years [M, SD]	20.9 (2.9)	20.8 (2.9)
Sociocultural risk factors	*N* (%)	*N* (%)
Mother’s age at child’s birth (in years)		
14–19	64 (10.7)	30 (46.9)
20–24	134 (22.5)	67 (51.0)
25–48	398 (66.8)	166 (41.7)
Mother’s schooling at child’s birth		
None	82 (13.8)	24 (29.3)
Primary	184 (31.0)	99 (53.8)
High school	169 (28.5)	91 (53.8)
Post-high school	159 (26.8)	49 (30.8)
Household income (in Thai Baht)		
0–49,999	216 (36.4)	102 (47.2)
50,000–99,999	116 (19.6)	69 (59.5)
100,000–199,999	129 (21.8)	52 (40.3)
200,000 plus	132 (22.3)	37 (28.0)
Religious affiliation		
Muslim and other	83 (14.0)	29 (34.9)
Buddhist	511 (86.0)	233 (45.6)
Single parent	216 (36.2)	94 (43.5)
Family size		
2–4	230 (38.6)	109 (47.4)
5–6	233 (39.1)	108 (46.4)
7 or more	133 (22.3)	45 (33.8)
Sex of child		
Female	298 (49.9)	115 (38.6)
Male	299 (50.1)	148 (49.5)
First child in family		
No	342 (66.0)	147 (43.0)
Yes	176 (34.0)	84 (47.7)

*M* Mean; *SD* Standard deviation

**Table 2:** Sample characteristics of overall sample (*n* = 597) and S-ECC sub-sample (*n* = 263)

Variables	All	S-ECC
	*N* (%)	*N* (%)
Risk behaviour		
Infant feeding at 6 months		
Never breast fed	295 (49.6)	145 (49.2)
Breast feeding: 1–3 months	155 (26.1)	55 (35.5)
Breast feeding: 4 months or more	145 (24.4)	62 (42.8)
Nocturnal feeding at 12 months		
Suckle to sleep when going to bed	563 (94.5)	251 (44.6)
Introduction of soft drinks at 12 months		
None	289 (49.1)	113 (39.1)
6–12 months	299 (50.9)	144 (48.2)
Sleeps with bottle at 30 months		
No	454 (76.2)	187 (41.2)
1–6 times/week	142 (23.8)	76 (53.5)
Sweet food index in days in a week at 30 months (1–5)	2.1 (1.3)	2.3 (1.2)
Brushing teeth in the past 2 weeks at 12 months	345 (58.3)	160 (46.4)
Brush with tooth paste at 12 months	10 (2.9)	3 (30.0)
Brushing teeth at 26 months		
At least once daily	160 (31.7)	147 (36.4)
Less than daily	345 (68.3)	84 (63.6)
Use of fluoride tooth paste in past 7 days	477 (88.8)	220 (89.8)
Use of oral health services		
Dental visit before age of 30 months	41 (6.9)	23 (56.1)

*GHQ* General Health Questionnaire

### Associations with severe early childhood dental caries

In bivariate analysis, environmental risk factors (the use of rain or well water as drinking water and secondary smoke), sociocultural risk factors (no schooling of mother of child, being male, lower household income, and being a Buddhist), and risk behaviours (never have breast fed the child, suckle to sleep when going to bed, introduction of soft drinks at 12 months, sleeps with the bottle at 30 months, having had more frequently sweet food, brushing teeth at 12 months and less than daily tooth brushing before 30 months) were associated with S-ECC. In multivariate logistic regression analysis, socio-environmental factors (the use of rain or well water as drinking water, no schooling of mother of child, being male), and risk behaviour (sleeping with a bottle at 30 months) were associated with S-ECC (see Table 3).

**Table 3:** Association between environmental, sociocultural, behavioural, dental care and severe early childhood caries

Variable	Severe early childhood caries	
	UOR (95 % CI)	AOR^a^ (95 % CI)
Environmental risk factors		
Drinking water in the household: Rain, well or other (base = Bottled or pipe)	1.30 (0.94–1.81)****	1.52 (1.00–2.33)*
Low birth weight (<2500 g) (base = ≥2500 g)	0.97 (0.53–1.78)	---
Height in cms at 6 months	1.01 (0.95–1.08)	---
BMI score at 3 years	0.98 (0.92–1.03)	---
Smoking during pregnancy (base = no) [too few cases]	1.24 (0.08–19.99)	---
Secondary smoke (at 1 year) (base = no)	1.26 (0.87–1.81)****	---
Mother with dental cavitation(s) at baseline (base = none)	1.05 (0.72–1.52)	---
Sociocultural risk factors		
Mother’s age at child’s birth (in years)		
14–19	1.00	---
20–24	1.13 (0.62–2.06)	
25–48	0.81 (0.48–1.38)	
Mother’s schooling at child’s birth		
None	1.00	1.00
Primary	2.82 (1.61–4.91)***	2.06 (1.24–3.42)**
High school	2.82 (1.61–4.95)***	2.21 (1.32–3.71)**
Post-high school	1.08 (0.60–1.93)	1.04 (0.45–2.54)
Household income (in Thai Baht)		
0–49,999	1.00	---
50,000–99,999	1.64 (1.04–2.59)*	
100,000–199,999	0.76 (0.49–1.17)****	
200,000 plus	0.44 (0.27–0.69)***	
Religious affiliation: Buddhist (base = Muslim and other)	1.56 (0.96–2.53)****	---
Single parent (base = not)	0.97 (0.69–1.36)	---
Family size	0.94 (0.88–1.01)	---
Sex of child: Male (base = female)	1.56 (1.13–2.11)**	1.68 (1.11–2.53)*
First child in family (base = no)	1.21 (0.84–1.75)	---
Risk behaviour		
Infant feeding at 6 months		
Never breast fed	1.00	1.00
Breast feeding: 1–3 months	0.57 (0.38–0.85)**	0.63 (0.38–1.04)
Breast feeding: 4 months or more	0.77 (0.52–1.15)****	
Nocturnal feeding at 12 months		
Suckle to sleep when going to bed	1.61 (0.77–3.38)****	---
Introduction of soft drinks at 12 months: 6–12 months (base = none)	1.45 (1.04–2.01)*	---
Sleeps with bottle at 30 months: 1–6 times/week (base = no)	1.64 (1.13–2.40)**	1.79 (1.10–2.92)*
Sweet food index in days in a week at 30 months: 3–7 times (base = 0–2 times)	1.23 (1.08–1.41)**	---
Brushing teeth in the past 2 weeks at 12 months (base = no)	1.25 (0.90–1.74)****	
Brush with tooth paste at 12 months (base = no)	1.20 (0.70–2.07)	
Brushing teeth at 26 months: Less than daily (base = At least once daily)	1.49 (1.02–2.17)*	
Brushing teeth at 26 months with fluoride tooth paste (base = no)	1.20 (0.70–2.07)	
Use of oral health services		
Dental visit before 30 months of age (base = no)	1.68 (0.89–3.18)	

*UOR* Unadjusted Odds Ratio; *AOR* Adjusted Odds Ratio, *CI* Confidence Interval

***P < 0.001; **P < 0.01; *P < 0.05; ****P ≤ 0.25

^a^Using *P* ≤ 0.25 as a selection criteria in univariate analysis; Backward conditional logistic regression method; Hosmer and Lemeshow Test: Chi-square = 4.23, *P* = 0.837; Nagelkerke *R* ^*2*^ = 0.10

## Discussion

The study found a high prevalence of severe caries at 36 months of age (44.1 %) in Northern Thailand, which is higher than in almost all previous studies on S-ECC [2–11]. In previous studies in Thailand also a very high prevalence of dental caries was found among children of 18 months (68.1 %) [25], and among preschool children (95.4 %) [26]. Less than 10 % of the sample had ever attended a dental visit before the age of 30 months. This may mean that that for a large group or pre-school aged children dental caries remained untreated. Increased preventive measures of the dental public health services targeting infants and pre-school children seem warranted, and early dental attendance, by 12 months of age, to enable anticipatory guidance of caries risk assessment and early management of dental problems [6].

The study investigated social factors for S-ECC in 3 year old the children.

In agreement with previous studies on S-ECC in pre-school children [2, 8, 10, 13, 14], this study found that lower socioeconomic status (no schooling of mother of child), risk behaviour (sleeping with a bottle at 30 months), and sub-optimal fluoridation of water supply (using drinking water from the rain in the household) were associated with S-ECC. Frequent sleeping with a bottle may increase caries development, since “low salivary flow rate during sleep time in infants decreases oral clearance and increases contact time of plaque and substrates.” ([4],p.139). Further, in agreement with a number of studies [13, 14, 16, 17] excessive sugar intake (introduction of soft drinks at 12 months, having had more frequently sweet food) were found in bivariate analysis associated with S-ECC. “Increased frequency of sugar consumption increases the risk of enamel demineralization and decreases time for remineralization by saliva.” ([4],p.139) Moreover, as found in previous studies, low-frequency tooth brushing and improper tooth brushing methods [4, 19] (less than daily tooth brushing before 30 months) were associated in bivariate analysis with S-ECC. An important preventive target should be to educate pregnant women or women with infants on avoidance of caries-promoting behaviours [2], such as the use of nursing bottles, excessive sugar intake and low-frequency toothbrushing and improper toothbrushing methods.

Unlike in some other studies [3, 11, 14, 17], this study did not find an association between the use of dental services, BMI overweight, being a single mother and maternal caries experience. After all, it may be useful to adopt an intervention approach, which considers underlying social determinants of dental health and utilizes a more client-centred approach, such as motivational interviewing [6].

### Study strength and limitation

This study was based on data from the prospective cohort study of Thai children (PCTC). Compared to a more traditionally designed study, this cohort study design with data collection at several time points during pregnancy and early childhood had the advantage to reduce recall bias [27]. Bias may have arisen in S-ECC and its social determinants between children who refused or were not present on the day for the dental examination (almost one-fourth of those who were recruited as pregnant women) and those children who were examined. It is known that children/parents refusing to participate may have higher caries prevalence than children who do participate in research studies, but it is probably no reason to assume that children who were not present at the day of examination differ from the participating children.

## Conclusion

A very high prevalence of S-ECC was found among pre-school children in Northern Thailand. Several risk factors, including environmental factors (sub-optimal fluoridation of water supply, no schooling of mother of child, being male), and risk behaviour (sleeping with a bottle at 30 months) were identified as possible risk factors for the development of S-ECC, which can help guide the development of prevention programmes of S-ECC for pre-school aged children.

## Abbreviations

BMI: Body Mass Index
DFMS: Decayed, missing, and filled surfaces
DMFT: Decayed, missing and filled teeth
PCTC: Prospective Cohort Study of Thai children
S-ECC: Severe Early Childhood Caries
